# Comparison of alphabetical versus categorical display format for medication order entry in a simulated touch screen anesthesia information management system: an experiment in clinician-computer interaction in anesthesia

**DOI:** 10.1186/1472-6947-12-46

**Published:** 2012-05-29

**Authors:** Anil A Marian, Franklin Dexter, Peter Tucker, Michael M Todd

**Affiliations:** 1 Department of Anesthesia, University of Iowa, 6JCP, 200 Hawkins Drive, Iowa City, IA 52242, USA; 2 Current Affiliation: Rush University, 600 S Paulina St, Chicago, IL 60612, USA

## Abstract

**Background:**

Anesthesia information management system (AIMS) records should be designed and configured to facilitate the accurate and prompt recording of multiple drugs administered coincidentally or in rapid succession.

**Methods:**

We proposed two touch-screen display formats for use with our department’s new EPIC touch-screen AIMS. In one format, medication “buttons” were arranged in alphabetical order (i.e. A-C, D-H etc.). In the other, buttons were arranged in categories (Common, Fluids, Cardiovascular, Coagulation etc.). Both formats were modeled on an iPad screen to resemble the AIMS interface. Anesthesia residents, anesthesiologists, and Certified Registered Nurse Anesthetists (n = 60) were then asked to find and touch the correct buttons for a series of medications whose names were displayed to the side of the entry screen. The number of entries made within 2 minutes was recorded. This was done 3 times for each format, with the 1^st^ format chosen randomly. Data were analyzed from the third trials with each format to minimize differences in learning.

**Results:**

The categorical format had a mean of 5.6 more drugs entered using the categorical method in two minutes than the alphabetical format (95% confidence interval [CI] 4.5 to 6.8, P < 0.0001). The findings were the same regardless of the order of testing (i.e. alphabetical-categorical vs. categorical - alphabetical) and participants’ years of clinical experience. Most anesthesia providers made no (0) errors for most trials (N = 96/120 trials, lower 95% limit 73%, P < 0.0001). There was no difference in error rates between the two formats (P = 0.53).

**Conclusions:**

The use of touch-screen user interfaces in healthcare is increasingly common. Arrangement of drugs names in a categorical display format in the medication order-entry touch screen of an AIMS can result in faster data entry compared to an alphabetical arrangement of drugs. Results of this quality improvement project were used in our department’s design of our final intraoperative electronic anesthesia record. This testing approach using cognitive and usability engineering methods can be used to objectively design and evaluate many aspects of the clinician-computer interaction in electronic health records.

## Background

An Anesthesia Information Management System (AIMS) has two components: (1) automatically validated data from the anesthesia machine and the physiological monitor and (2) the data manually entered by the anesthesia provider including events, medications, information on airway management, etc. Anesthesia residents, anesthesiologists, and Certified Registered Nurse Anesthetists (anesthesia providers) using AIMS need to record the manual data while simultaneously engaged in other more vital tasks. One example involves medications: an anesthesia provider may make dozens of drug-dose entries within a few hours. Typically, no pharmacist or nurse is involved in medication delivery. The anesthesia provider decides on the drug, draws it up, and administers it, often within seconds or minutes. The rate of required drug data-entry is not uniform over time. Many more entries are required during the relatively short period of induction and other critical phases than during maintenance. A well-designed AIMS should facilitate the accurate and rapid recording of multiple drugs.

Our hospital made the decision to implement EPIC’s AIMS (Epic Systems, Verona, WI). This system allows the user to configure many of its components - including drug entry formats. During our development period, we performed a systematic search for experimental and observational studies of the impact of display format on the rate of entry of medications (and on errors) and found none for any specialty.^a^ We therefore undertook a study to determine which of two basic entry formats was better for fast and accurate data entry. We evaluated how design variation (alphabetical versus categorical) and user variation (users with different levels of clinical experience) affects the task and the occurrence of errors.

## Methods

This project was performed as a quality improvement project. No records were maintained of the names of any participants. Results were used for the design of our final AIMS order entry screen. The University of Iowa Institutional Review Board reviewed our application for publication of the results and concluded “this is not human subjects research.”

Two touch-screen display formats were programmed on an iPad (Apple Inc., Cupertino, CA) to resemble the proposed AIMS medication order entry touch screens. The iPad program was Web-based, utilizing ASP.net, jQuery and SQL Server. Each time a participant selected a drug, the server recorded the result of that selection. Each trial lasted 2 minutes. The number of entries completed within the 2 minutes was recorded, along with any entry errors.

**Figure 1:**
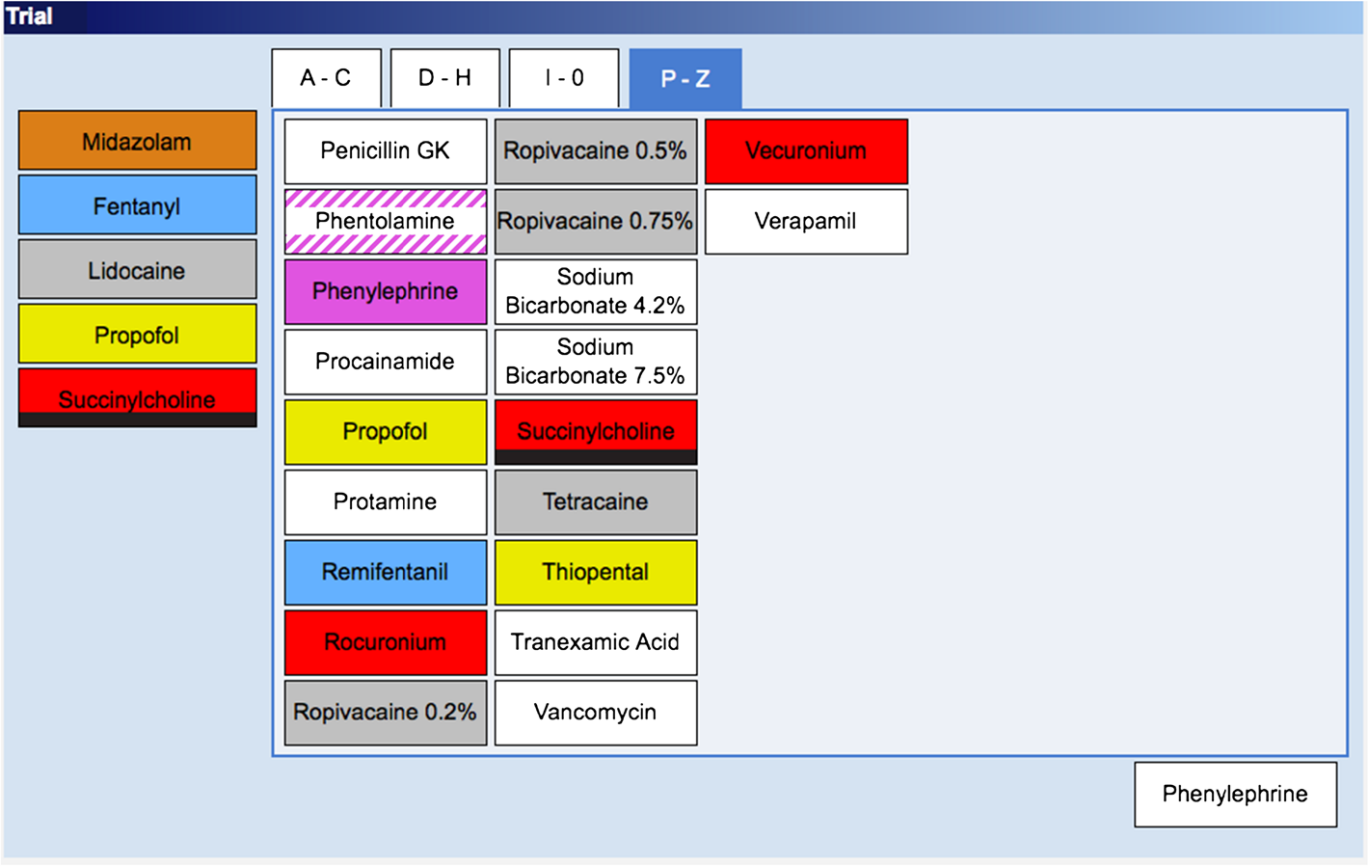
**Screen shot of alphabetical display format.** This drug entry screen was constructed with "tabs" across the top labeled A-C, D-H, I-O and P-Z. Under each tab, individual drugs were listed in alphabetical order.

**Figure 2:**
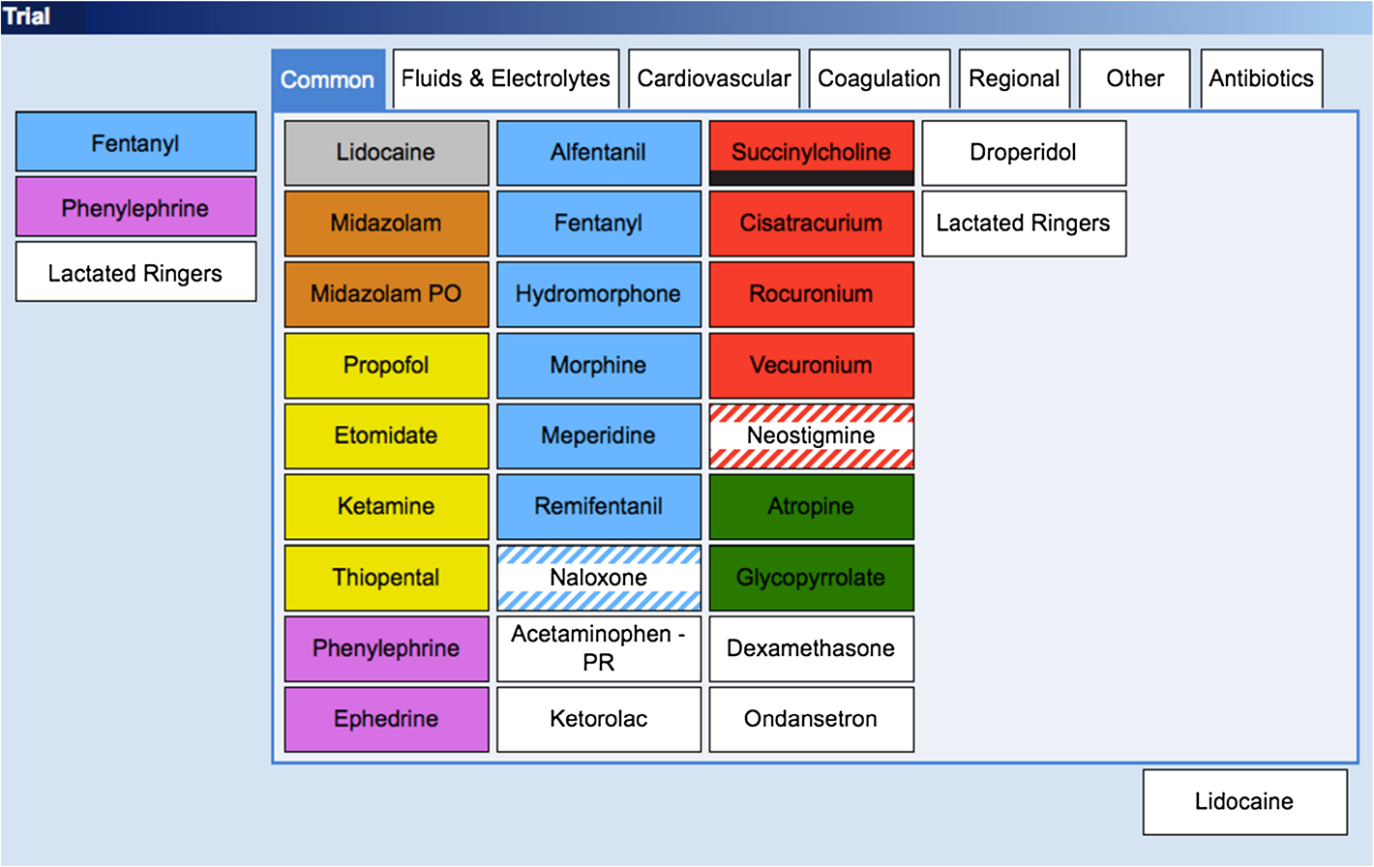
**Screen shot of categorical display format.** The drug entry screen was constructed with “tabs” across the top for Common Drugs, Fluids and Electrolytes, Cardiovascular, Coagulation, Regional Anesthetic, Antibiotics and Other Drugs. Under each category, drugs were grouped by category.

The two formats were alphabetical and categorical. For the first, the entry screen was constructed with “tabs” across the top labeled A-C, D-H, I-O and P-Z. Under each tab, individual medication “buttons” were listed in alphabetical order (e.g. acetaminophen, albuterol, alfentanil, atropine etc.). There were roughly 25–30 medications under each alphabetical tab. For categorical format, the entry screen was constructed with tabs across the top for Common Drugs, Fluids and Electrolytes, Cardiovascular, Coagulation, Regional Anesthetic, Antibiotics and Other Drugs. Under each category, drugs were grouped by category (e.g. all neuromuscular blocking drugs together, all opioids together etc.) Drugs listed under Common Drugs were chosen based on an extensive review of anesthetic records, and represented the 29 medications used most commonly in our operating rooms. Colors were used to label medications to match American Society for Testing and Materials (ASTM) standards as much as possible. Screen shots of example screens for both formats are shown in Figures 1 and 2.

There were 132 medications included in each of the study formats.

The study was conducted in the operating room environment but not during periods of patient care. Three groups of participants were chosen, based on their years of clinical anesthesia experience: anesthesia residents and student registered nurse anesthetists (SRNA) with <1 year of clinical anesthesia experience; anesthesia residents, fellows, and SRNA with 1 to 3 years of anesthesia experience; and anesthesiologists and Certified Registered Nurse Anesthetists, all with ≥ 4 years of experience. The convenience samples were N = 20 participants per group, selected by the first author. Everyone invited to participate chose to do so. Each participant participated in the study individually, not in groups.

### Testing protocol

Participants were handed the iPad, which displayed a brief introduction of the study and confirmed their willingness to participate in the study. Each participant entered his or her years of clinical experience. Successive participants in each group were assigned in alternating order to an initial format, either alphabetical or categorical. Next, the participants underwent a training exercise to familiarize them with the system. The participants were presented with the names of countries, grouped either alphabetically or categorically by continent based on the initial assignment. The participant was prompted with the name of a country in the lower right corner of the screen and was then required to locate that country under one of the tabs, and tap the button for that country. If correct, the country moved automatically to the left side of the screen in a vertical list. A different country name then replaced the current country in the lower right portion of the screen. The demonstration ended when the participant successfully tapped four countries.

At the end of this training period, the participant tapped the Start Trial button. Based on the assignment either the alphabetical or categorical format appeared. The name of a drug appeared on the lower right corner of the screen and the participant was required to find that drug under the various tabs and touch the correct button to complete its entry. When the correct drug was selected, a new drug name would appear on the screen. Participants had 2 minutes to find and enter as many drugs as possible from a list of 25 drugs that were displayed in an order (Table 1). Should they complete the 25 drugs within the allotted time, the list would cycle back to the first drug. At the end of the 2 minutes trial, they were shown their results (number of drug entered, number of errors) and directed to begin the next trial. The second trial used the same format and requested the entry of the same drugs as the first trial; however, the order with which drugs were listed was changed. This same process was repeated for a third trial. Participants were next instructed that the format would change from the previously viewed alphabetical format to a categorical format, or vice-versa. They repeated the demonstration with countries using the new format. They then completed an additional three 2 minute long drug-entry trials using the new format.

**Table 1:** Order of Drugs in Trial 1, Trial 2 and Trial 3

**Trial 1**	**Trial 2**	**Trial 3**
Midazolam	Fentanyl	Lidocaine
Fentanyl	Phenylephrine	Cefazolin
Lidocaine	Lactated Ringers	Bupivacaine 0.25%
Propofol	Lidocaine	Midazolam
Succinylcholine	Epinephrine	Heparin
Phenylephrine	Propofol	Propofol
Rocuronium	Succinylcholine	Lactated Ringers
Ephedrine	Ephedrine	Succinylcholine
Lactated Ringers	Rocuronium	Albumin 5%
Dexamethasone	Cefazolin	Rocuronium
Fentanyl	Rocuronium	Fentanyl
Cefazolin	Albumin 5%	Insulin
Albumin 5%	Insulin	Fentanyl
Epinephrine	Dexamethasone	Epinephrine
Rocuronium	Fentanyl	Dexamethasone
Norepinephrine	Norepinephrine	Norepinephrine
Insulin	Midazolam	Ondansetron
Heparin	Heparin	Morphine
Ketorolac	Bupivacaine 0.25%	Ketorolac
Morphine	Glycopyrollate	Phenylephrine
Lactated Ringers	Lactated Ringers	Lactated Ringers
Ondansetron	Neostigmine	Rocuronium
Neostigmine	Morphine	Fentanyl
Glycopyrollate	Ondansetron	Neostigmine
Bupivacaine 0.25%	Ketorolac	Glycopyrollate

The lists of 25 drugs were created from the 22 commonly used anesthestic drugs in our department. The list repeated from the top if/when a clinician had entered all 25 drugs within 2 minutes and had time remaining. Because the list contains 25 drugs and yet 22 drugs were used often, there are duplicates within the lists: Fentanyl, Lactated Ringers, and Rocuronium.

The drugs were displayed in the same sequence for corresponding trials between the different formats (i.e. the sequence of drugs was the same for Trial 4 as it was for Trial 1). The total time for each participant was approximately 20 minutes.

### Analysis

To avoid any problems with a “learning curve,” only the number of drugs entered on the third trial with each format was used in our primary analysis. Numbers of drugs entered in each 2 minute period were compared pairwise by participant using two-sided one-group Student’s t tests. The pairwise differences were reported as mean ± standard deviation. Analyses were repeated after stratifying by the initial format viewed, alphabetical or categorical. Those differences were consistent with normal distributions (Shapiro-Wilk P > 0.05).

Statistical analyses of the numbers of medications entered incorrectly were performed using two-group permutation tests. This test is like the Wilcoxon signed-ranks test, but includes the 0’s. The zeros mattered because most participants had no errors for most trials. Confidence intervals for median differences were calculated using the Hodges-Lehman method. All results were calculated using exact methods (StatXact-9, Cytel Software Corporation, Cambridge, MA).

## Results

Providers using the categorical format had a mean of 32.5 drugs entered in two minutes in the third trial compared to 26.8 drugs in the alphabetical format. The mean pairwise difference was 5.6 ± 4.4 more drugs entered using categorical than alphabetical format (95% confidence interval 4.5 to 6.8, P < 0.0001). The order of testing did not alter this result. Individuals tested first with the alphabetical format averaged 6.0 ± 4.8 more drugs entered by category (95% CI 4.2 to 7.7, P < 0.0001) while those tested first with the categorical format averaged 5.3 ± 4.0 more drugs entered via the categorical format (95% CI 3.8 to 6.8, p < 0.0001). A similar pattern was observed when providers were analyzed by years of experience.

**Figure 3:**
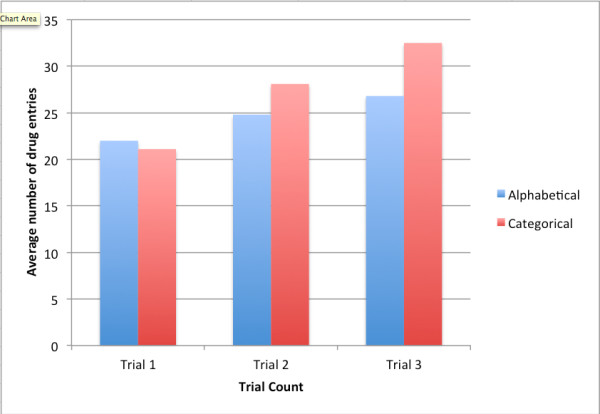
**Bar Chart showing the average number of drugs entered in the alphabetical and categorical formats during Trial 1, Trial 2 and Trial 3.** The x-axis shows the trial number and the y-axis shows the number of drugs entered in each trial. Only the number of drugs entered in Trial 3 was used in the primary analysis to avoid any problems with “learning curve”. The magnitude of the learning curve was estimated by taking for each participant the average of two pairs of numbers of drugs entered: (Trial 3 categorical – Trial 1 categorical) and (Trial 3 alphabetical – Trial 1 alphabetical). The mean ± standard deviation among participants of the average differences were 8.1 ± 3.4 drugs entered (P < 0.0001 in comparison to 0; N = 60).

Since categories rely on learning, a learning effect was expected and observed. For the first trial, alphabetical was similar to categorical, providing 0.9 ± 5.5 more drugs entered than categorical (95% CI −0.6 to 2.3, P = 0.23). By the third trial, the categorical format resulted in 5.6 ± 4.4 more drugs entered in two minutes (Figure 3).

Most anesthesia providers made no (0) errors for most trials (N = 96/120 trials, lower 95% limit 73%, P < 0.0001). There was no difference in error rates between alphabetical and categorical templates at both the first (P = 0.54) and third trials (P = 0.53). The corresponding 95% confidence intervals for the median differences were 0 to 0 and 0 to 1 (categorical less), respectively.

## Discussion

The designing and layout of the user interface of an electronic medical record can influence task load, time to task completion, and number of errors of cognition associated with the identification, and subsequent use, of relevant patient data by the clinical provider [1]. Among US anesthesiologists using AIMS, 40% surveyed identified less efficient workflow as a problem with AIMS [2]. With a paper record, writing down the name of a drug and dose administered takes a couple of seconds. With a touch screen interface, the anesthesia provider has to find the drug from the list of available (e.g., 132) medications and document it.

Cognitive demands introduced by the system on physicians who are already engaged in many other vital tasks contribute to decrease in efficiency [3]. Horsky and colleagues developed a methodology for the characterization of cognitive demands of a medical information system, which was based on the distributed resources model, an approach that describes the dimensions of user interfaces that introduce unnecessary cognitive complexity. Semantic matching rather than alphanumeric ordering or strict hierarchies may expedite searches for orders, sets, and text-based values in pick lists that frequently contain dozens of items [4].

Human–computer interaction has been an important part of cognitive science for decades [5]. *Usability* of a computer system is defined as the capacity of the system to allow users to carry out their tasks safely, effectively and efficiently [6]. *Usability testing* refers to the evaluation of information systems that involves testing of participants who are representative of the target population [7]. Our study resulted in usability testing of the medication order entry screen of touch screen AIMS by our anesthesia providers. Cognitive task analysis (CTA) for evaluation of medical systems represents the integration of work from the field of systems engineering and cognitive research in medicine [7]. In our study, CTA involved the effect of design variation and user variation in the completion of task and error rate in an AIMS.

Our experimental study had multiple limitations. First, this project was performed as a quality improvement project, using a convenience sample of available clinicians, with no formal power analysis performed until there were N = 60 participants at which time the project was stopped based on the findings. Second, the 22 medications studied are those commonly used in our department (Table 1). Results may be different for departments routinely using fewer or greater numbers of medications. Third, we did not include interruptions or distractions. Although we do not expect that results would be different when an anesthesia provider is interrupted in the middle of entering a series of medications, our experiment was not designed to investigate that possibility. Fourth, even though faster may not mean better, speed of entry along with the number of errors was chosen as a reasonable surrogate for ease of use. Fifth, we only studied the entry of a drug name, not the dose, even though designing of the interface can have a significant impact on the drug dosing errors in a health information system [8,9]. Sixth, we used an AIMS medication entry screen virtually identical to the actual screen in Epic. Whether or not our conclusions can directly be extrapolated to another AIMS - perhaps one based on keyboard or mouse-based entry - is unclear, although clearly the same testing protocol could be developed and used for other systems.

## Conclusion

**Figure 4:**
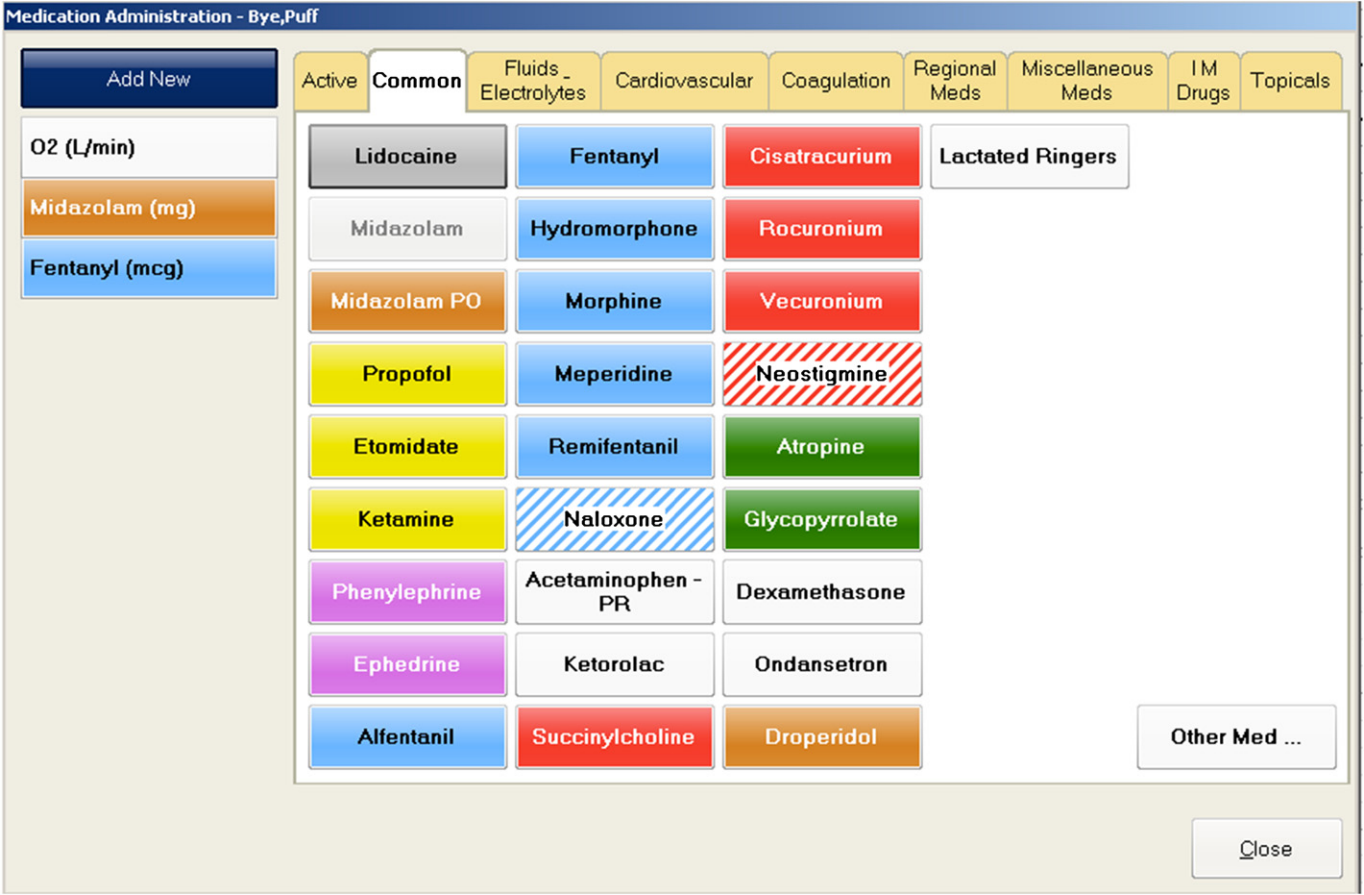
**Final Anesthesia medication order entry screen that was implemented in the anesthesia information management system based on the study.** This figure can be compared to the experimental Figure 2.

Arrangement of data entry elements in a logical fashion is an important feature of design of electronic medical records. Our results show that the arrangement of drugs in a categorical format in the medication order-entry screen of an AIMS can result in faster data entry compared to an alphabetical arrangement of drugs. Results of this quality improvement project were used in our department’s design of our final intraoperative electronic anesthesia record (Figure 4).

## Endnotes

^a^The following PubMed search identified 26 articles September 20, 2010: ( medical order entry systems[MeSH] OR order entr*[TIAB] OR drug list*[TIAB] or medication list*[TIAB] OR anesthesia information[TIAB] ) AND ( alphabet*[TIAB] OR categor*[TIAB] ) AND ( experiment*[TIAB] OR laborator*[TIAB] OR participant*[TIAB] OR random*[TIAB] OR simulat*[TIAB] OR subject*[TIAB] ). No article was relevant in terms of comparing alphabetical versus categorical drug lists. When repeated December 18, 2010, there were 31 articles identified. One is an experimental study evaluating the impact of interruptions on drug entry errors [10].

## Competing interests

The authors declare that they have no competing interest.

## Authors’ contributions

AAM designed the study, conducted the study, analyzed the data and wrote the manuscript. FD designed the study, analyzed the data and wrote the manuscript. PT designed the study and conducted the study. MMT designed the study and wrote the manuscript. All authors read and approved the final manuscript.

## Pre-publication history

The pre-publication history for this paper can be accessed here:

http://www.biomedcentral.com/1472-6947/12/46/prepub
